# Elevated Epicardial Adipose Tissue and Ischemic Stroke Risk: A Systematic Review and Meta-Analysis †

**DOI:** 10.3390/medicina61122128

**Published:** 2025-11-28

**Authors:** Arankesh Mahadevan, Monitha Pinnamaneni, Manaswini Krishnakumar, Tanisha Mishra, Parth Adrejiya, Aditya Sanjeevi, Bhaumikkumar Mukeshbhai Patel, Sneh Patel, Rahul Patel, Nihar Jena, Ankit Vyas, Rupak Desai

**Affiliations:** 1 Department of Neurology, University of Utah Health, Salt Lake City, UT 84132, USA; arankeshmahadevan@gmail.com; 2 Department of Internal Medicine, The Christ Hospital, Cincinnati, OH 45219, USA; monithap@gmail.com; 3 Department of Internal Medicine, Saint Vincent Hospital, Worcester, MA 01608, USA; manas.krish97@gmail.com; 4 Department of Internal Medicine, University of Connecticut School of Medicine, Farmington, CT 06030, USA; tmishra1001@gmail.com; 5 Department of Internal Medicine, Wellstar Spalding Medical Center, Griffin, GA 30224, USA; parthadrejiya@gmail.com; 6 Department of Internal Medicine, Rochester General Hospital, New York, NY 14621, USA; aditya.sanjeevi7@gmail.com; 7 Department of Internal Medicine, M. P. Shah Medical College, Jamnagar 361006, India; drbpatel1996@gmail.com; 8 Department of Internal Medicine, Mercy Hospital Fort Smith, Fort Smith, AR 72903, USA; patelsneh32@gmail.com; 9 Department of Internal Medicine, UNC Health Blue Ridge, Morganton, NC 28655, USA; rahulpatel21295@gmail.com; 10 Interventional Cardiology, WVU/Camden Clark Medical Center, Parkersburg, WV 26101, USA; 11 Department of Vascular Medicine, Ochsner Clinic Foundation, Jefferson, LA 70121, USA; drankitgvyas@gmail.com; 12 Independent Researcher, Outcomes Research, Atlanta, GA 30033, USA; drrupakdesai@gmail.com

**Keywords:** atrial fibrillation, ischemic stroke, epicardial adipose tissue, risk stratification

## Abstract

*Introduction:* Epicardial adipose tissue (EAT), a fat depot between the myocardium and pericardium, produces pro-inflammatory adipokines, contributing to inflammation, insulin resistance, and endothelial dysfunction. EAT has been recognized as an independent risk factor for cardiovascular diseases, including atrial fibrillation (AFib) and acute ischemic stroke (AIS). This study explores the association between EAT and AIS risk, with a focus on populations with cardiovascular comorbidities. *Material and Methods*: This meta-analysis adhered to MOOSE and PRISMA guidelines. A comprehensive search of PubMed, SCOPUS, and Embase databases was conducted, targeting studies evaluating the association between EAT and AIS. Inclusion criteria encompassed RCTs, cohort, case–control, and cross-sectional studies. Quality assessment was performed using appropriate tools, and statistical analysis involved pooled odds ratios (ORs) with 95% confidence intervals (CIs) using a binary random-effects model. *Results:* The search identified 711 studies, eight of which met the inclusion criteria, yielding 7412 participants. Analysis revealed that increased EAT thickness significantly correlated with higher odds of AIS (aOR: 3.60 [2.26–5.74], I^2^ = 74.24%). Sensitivity analysis confirmed the robustness of these findings despite publication bias. Higher epicardial adipose volume was also associated with an increased AIS risk (aOR: 1.17 [1.03–1.34], I^2^ = 49.54%). *Conclusions:* Increased EAT thickness and volume are associated with a higher risk of AIS in populations with cardiovascular comorbidities, including AFib. EAT’s pro-inflammatory and pro-thrombotic properties may contribute to stroke pathophysiology. These findings highlight the potential utility of EAT measurement in stroke risk stratification and support further research to integrate EAT assessment into clinical practice.

## 1. Introduction

Identifying risk factors for acute ischemic stroke (AIS) is critical due to its substantial healthcare and economic burden. From the 2016 Global Burden of Disease study, stroke accounted for 5.5 million deaths and 116.4 million disability-adjusted life years (DALYs) [1]. There is emerging interest in the association between regional fat depots and stroke, with Rodríguez-Granillo et al. identifying higher periaortic fat attenuation to be associated with cardioembolic strokes [2].

The association between epicardial adipose tissue (EAT) and AIS risk is a similar emerging area of interest, given the established links between EAT and various cardiovascular diseases [3]. EAT, a metabolically active fat depot surrounding the heart, is implicated in the pathogenesis of cardiovascular conditions due to its pro-inflammatory properties. It has been shown to originate from a similar embryonic tissue as omental and mesenteric fat, producing cytokines similar to abdominal visceral fat, leading to inflammation, insulin resistance, and endothelial dysfunction [4,5,6,7].

Atrial Fibrillation (Afib) is a well-established risk factor for stroke, and literature supports that EAT likely indirectly increases stroke risk by promoting Afib. As studies have reported higher EAT to be associated with adverse cardiovascular outcomes in patients with Afib and stroke risk after Afib ablation [8,9]. Despite the growing evidence linking EAT to cardiovascular outcomes and its established role in promoting AFib—a major contributor to stroke—the direct association between EAT and acute ischemic stroke (AIS) risk remains underexplored.

Prior meta-analyses have largely focused on the relationship between EAT and composite cardiovascular outcomes such as myocardial infarction, heart failure, and coronary artery disease, without isolating cerebrovascular endpoints. Consequently, the independent contribution of EAT to ischemic stroke risk remains uncertain. Moreover, no prior synthesis has systematically compared EAT thickness and volumetric measurements. This gap underscores the need for a systematic evaluation of the available evidence to clarify the role of EAT in AIS risk. Our pooled analysis aims to address this need by synthesizing data to determine whether EAT contributes to stroke risk, providing insights that could inform risk stratification and preventive strategies in clinical practice. To our knowledge, this is the first meta-analysis focused exclusively on stroke outcomes, and it is the only synthesis that models both EAT thickness and volume.

## 2. Materials and Methods

This meta-analysis was conducted in accordance with the Meta-Analysis of Observational Studies in Epidemiology (MOOSE) statement and adhered to the Preferred Reporting Items for Systematic Reviews and Meta-Analyses (PRISMA) guidelines to ensure methodological rigor, transparency, and reproducibility. As the study synthesized data from previously published research without direct involvement of human participants or primary data collection, institutional ethical approval and informed consent were not required. Registration and Protocol: This review was not prospectively registered in any registry, and no formal review protocol was prepared or published.

### 2.1. EAT Measurement and Imaging Modalities

Several imaging modalities, including echocardiography, computed tomography (CT), and magnetic resonance imaging (MRI), are used to estimate EAT volume, each with unique strengths and limitations. Echocardiography, a non-invasive and accessible method, measures EAT thickness well but lacks accuracy for volumetric estimates. Despite this, EAT thickness on echocardiography correlates with adverse outcomes like myocardial infarction and atrial fibrillation [3]. CT, the gold standard for EAT quantification, offers high spatial resolution and reproducibility, with EAT volume linked to cardiovascular risks such as coronary artery disease [10]. MRI, a radiation-free alternative, provides comprehensive volumetric analysis through 3D slice summation [11].

### 2.2. Search Strategy and Data Sources

A clearly defined research question was developed and structured according to the PECO (Population, Exposure, Comparison, Outcome) framework, focusing on the relationship between epicardial adipose tissue (EAT) and ischemic stroke. Specifically, the objective was to assess whether individuals with elevated EAT compared to those with low or normal EAT have a higher risk of developing ischemic stroke.

To address this question, two independent physician investigators conducted a comprehensive and systematic literature search across three major electronic databases: PubMed, SCOPUS, and Embase. The search strategy was designed to maximize sensitivity and specificity by combining free-text keywords and controlled vocabulary terms (Medical Subject Headings, MeSH) relevant to EAT and ischemic stroke. The exact search criteria used was ((((((((“Epicar*” [tiab] OR “pericar*” [tiab]) AND (“adipose” [tiab] OR “fat” [tiab])) OR “epicardial adipose” [tiab] OR “pericardial adipose” [tiab] OR “epicardial fat” [tiab] OR “pericardial fat” [tiab]) AND (“Stroke” [Mesh] OR “cerebrovascular” [tiab] OR “stroke” [tiab] OR “brain ischemia” [tiab] OR “thromboly*” [tiab] OR “tpa” [tiab] OR “thrombect*” [tiab] OR “brain infarct*” [tiab] OR “cerebral ischemi*” [tiab])) NOT (“review” [Publication Type])) NOT (“systematic review” [Publication Type])) NOT (“meta analysis” [Publication Type])) NOT (“case reports” [Publication Type])) NOT (“comment” [Publication Type]). The complete and detailed search strategy is provided in the Supplementary Materials for transparency and reproducibility (Supplemental Table S1).

Following the initial database queries, titles and abstracts were screened in duplicate for relevance, with disagreements resolved through discussion and, if necessary, by consultation with a third reviewer. Eligible studies were then assessed in full text against predefined inclusion and exclusion criteria.

### 2.3. Study Selection and Inclusion Criteria

We included randomized controlled trials (RCTs), cohort studies (prospective or retrospective), case–control studies, or cross-sectional studies evaluating the association between EAT and stroke from inception till April 2024. Only studies published in English involving patients aged 18 years or older were included, whereas preclinical and animal studies were excluded from the analysis. Studies with insufficient data or reviews without original data, abstracts and unpublished studies were also excluded. Following a database search, studies meeting inclusion criteria underwent forward citation and backward reference searches using the CitationChase software 1.11 suite to identify additional studies that would meet our above inclusion criteria [12]. Two independent reviewers extracted data from all eligible studies. Any discrepancies between the two reviewers were resolved through discussion and, when necessary, adjudicated by a third investigator.

### 2.4. Quality Assessment

The methodological quality and risk of bias of included studies were systematically evaluated. For non-randomized observational cohorts and case–control studies, we applied the Newcastle–Ottawa Scale (NOS), which assesses studies across three domains: selection of participants, comparability of cohorts, and ascertainment of exposure and outcomes. For randomized controlled trials (RCTs), we used the Joanna Briggs Institute checklist for randomized controlled trials (JBI-RCTs), which evaluates aspects such as randomization, allocation concealment, blinding, and completeness of outcome data.

**Figure 1:**
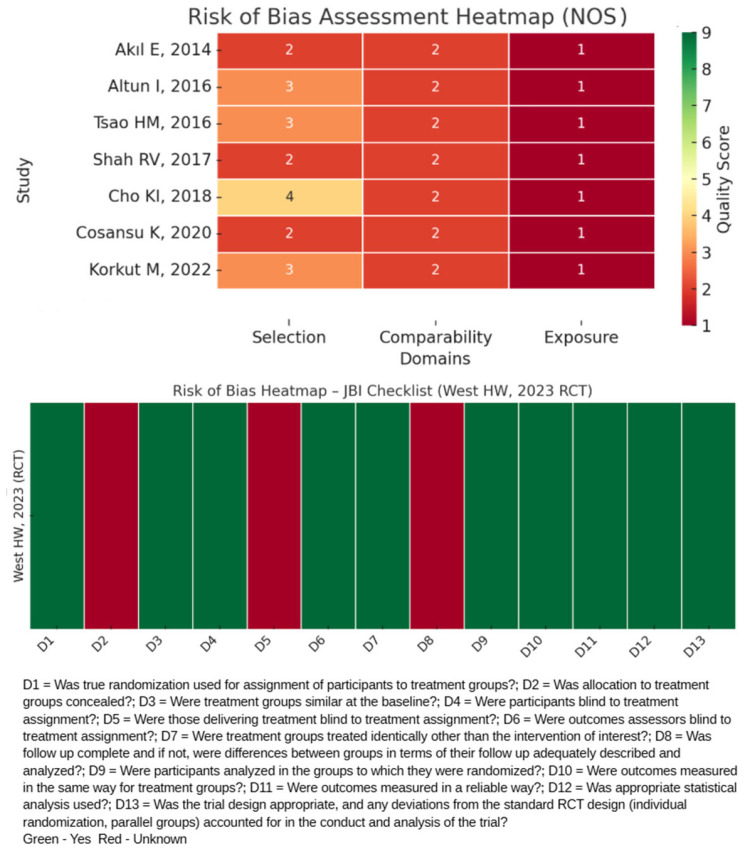
Risk of Bias Heatmap using Newcastle–Ottawa Scale and JBI Checklist for Randomized Controlled Trials.

Three reviewers independently performed the risk of bias assessments to minimize subjectivity. Inter-rater reliability was monitored throughout the process, and any disagreements were resolved through group discussion, followed by consensus with input from all senior authors when necessary. Study-level NOS and JBI-RCT scores are summarized in Table 1, with the distribution of risk (low, moderate, and high) visually illustrated in Figure 1. Sensitivity analyses were performed, excluding studies with moderate or high risk of bias to assess the robustness of pooled effect estimates and confirm the overall association between elevated EAT and AIS risk to support the reliability of the findings.

### 2.5. Statistical Analysis

Our meta-analysis utilized pre-calculated unadjusted and adjusted odds ratios (ORs) with 95% confidence intervals (CIs) to measure the association between EAT and the odds of AIS. We assessed publication bias using the Luis Furuya-Kanamori (LFK) index, illustrated in a DOI plot [21]. A binary random-effects model estimated the pooled OR for the meta-analysis, and the results were presented in forest plots. A *p*-value < 0.05 defines statistical significance. Pooled odds ratios (ORs) with 95% confidence intervals were computed with a DerSimonian–Laird random-effects model using the Meta-Analysis command set in IBM SPSS Statistics v29. Between-study heterogeneity was quantified with the I^2^ statistic and Q test. Potential sources of heterogeneity, including study design (case–control vs. cohort), imaging modality (CT vs. echocardiography), and measurement type (EAT thickness vs. volume), were explored qualitatively by comparing study-level characteristics. Robustness of the summary estimate was explored with a leave-one-out sensitivity analysis. No imputation of missing summary statistics was performed. When effect estimates were reported in different formats (e.g., hazard ratios or risk ratios), they were converted to odds ratios (ORs) for uniformity where appropriate, using standard formulae. Study-level characteristics were summarized in tabular form, detailing study design, population characteristics, EAT definitions, and outcomes [Table 2].

Publication bias and small-study effects were assessed both visually using funnel plots and quantitatively using the Luis Furuya-Kanamori (LFK) index, which was illustrated with a DOI plot for enhanced interpretability. Consistent with Cochrane methodological guidance, meta-regression was not attempted, as fewer than ten studies were available, thereby limiting statistical power and increasing the risk of type I error.

## 3. Results

### 3.1. Systematic Search and Quality Assessment

**Figure 2:**
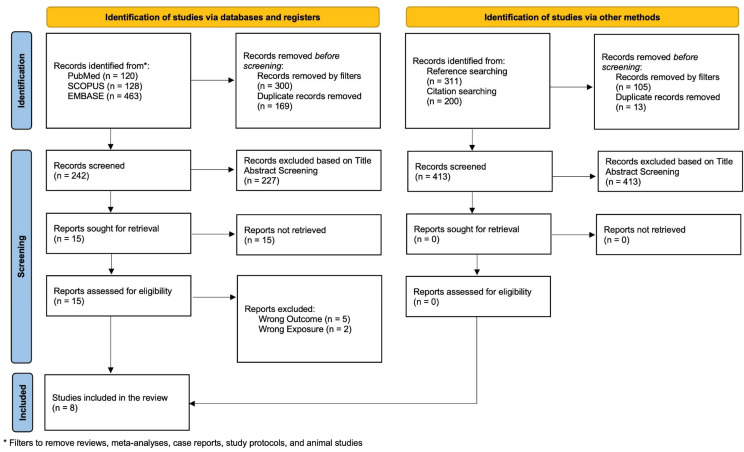
PRISMA Flow Diagram of the Systematic Search and Screening Process.

Our search across PubMed, SCOPUS, and EMBASE databases yielded 711 studies initially. Of these, 469 were removed as they were recognized as duplicates, and the remaining studies were filtered to include only articles with original data. Non-English and animal studies were also removed. We employed a double-reviewer blinded title and abstract screening process from which 227 of 242 articles were excluded. The subsequent 15 studies underwent full-text review, with seven excluded due to wrong outcomes and exposure criteria. Following the database search, the eight identified studies underwent hand-reference searching and forward citation searching using the CitationChaser software tool, resulting in a pool of 413 studies for evaluation. After removing 118 studies for duplicates and filtering, none of the 295 studies met our inclusion study criteria upon screening, and none met our finalized inclusion criteria. In summary, eight full-text studies were deemed eligible for inclusion in our meta-analysis and are reported as per the PRISMA flowchart (Figure 2).

An overview of the included studies is expanded in Table 2. After identifying eligible studies, the risk of bias assessment was conducted using the NOS and JBI-RCT as appropriate. The NOS evaluates the methodological quality of non-randomized observational studies by assessing selection, comparability, and outcomes domains, while the JBI-RCT tool, developed by the Joanna Briggs Institute, is designed to critically appraise the methodological quality of randomized controlled trials, focusing on domains such as randomization, allocation concealment, blinding, and the integrity of statistical analysis. Our analysis revealed that seven studies displayed a moderate risk of bias, while one exhibited a low risk of bias (Table 1).

### 3.2. Epicardial Adipose Thickness and Acute Ischemic Stroke

**Figure 3:**
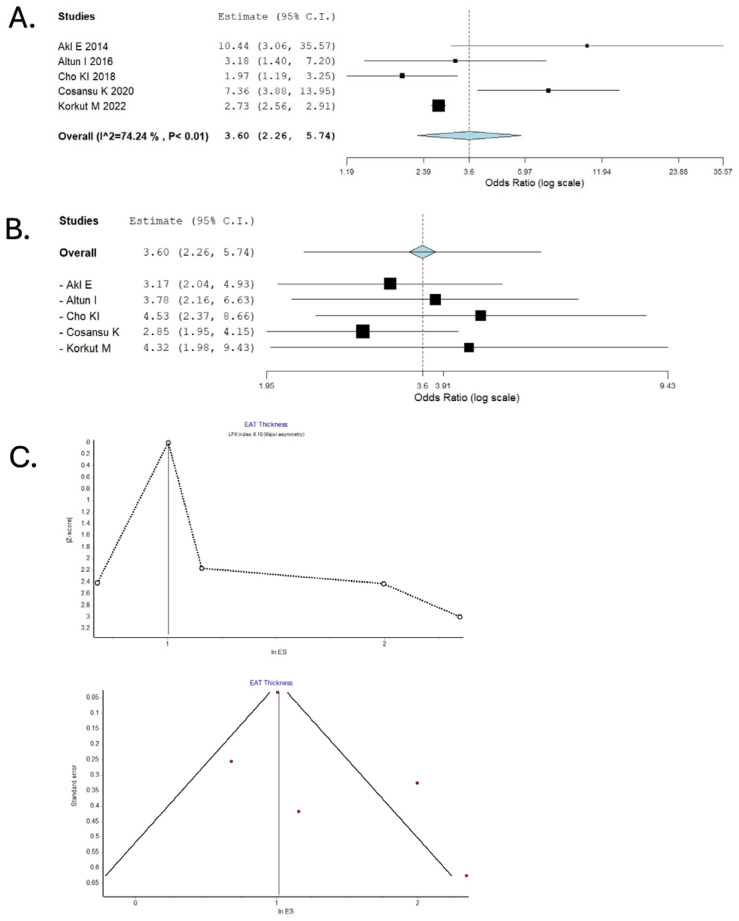
Association Between Epicardial Adipose Tissue Thickness and Risk of Ischemic Stroke: Pooled Analysis and Assessment of Publication Bias. (**A**) Forest plot depicting random-effects model pooled data for the association between high vs. low EAT thickness and risk of ischemic stroke. (**B**) Sensitivity analysis by the leave-one-out method. (**C**) Publication bias assessed using DOI and Funnel Plots. In the forest plots, the size of each black square represents the weight of the corresponding study in the meta-analysis (larger squares indicate greater statistical weight). The center of each square denotes the study-specific effect estimate, and the horizontal line represents its 95% confidence interval. Red squares indicate the pooled effect estimate in sensitivity analyses.

Our analysis exploring the association between EAT thickness and AIS identified a total of 5 studies, including two cross-sectional studies (Akıl E, Cosansu K), one retrospective (Cho KI), and two prospective studies (Altun I, Korkut M) from the years 2014 and 2022, and included a total of 696 patients [14,17,18,19,21]. The studies by Cho Kl and Consasu K et al. specifically included atrial fibrillation patients. Our pooled analysis found the adjusted odds ratio (aOR) to be 3.60 [2.26–5.74] with high heterogeneity (I^2^ = 74.24%, *p* < 0.01). Sensitivity analysis was performed, initially omitting the study by Akil E et al. due to it appearing to be an outlier (aOR: 10.44 [3.06–35.57]), and our findings remained significant (aOR: 3.17 [2.04–4.93]). Our findings remained robust even after excluding Korkut et al., which had the highest weightage to our pooled results (aOR: 4.32 [1.98–9.43]). Visual inspection of the funnel and DOI plots shows asymmetry, and the LFK index of 6.1 suggests major asymmetry, suggesting that our results may be confounded by publication bias [Figure 3]. This major asymmetry implies that smaller studies with null or negative findings may have been selectively withheld from publication, leading to an overestimation of the true pooled effect size for EAT thickness. The implication for clinical significance is that the modest odds ratio we report may be inflated. Most studies contributing to this synthesis were rated as having a moderate risk of bias, primarily due to participant selection and lack of blinding, which may modestly influence the pooled estimate.

### 3.3. Epicardial Adipose Volume and Acute Ischemic Stroke

**Figure 4:**
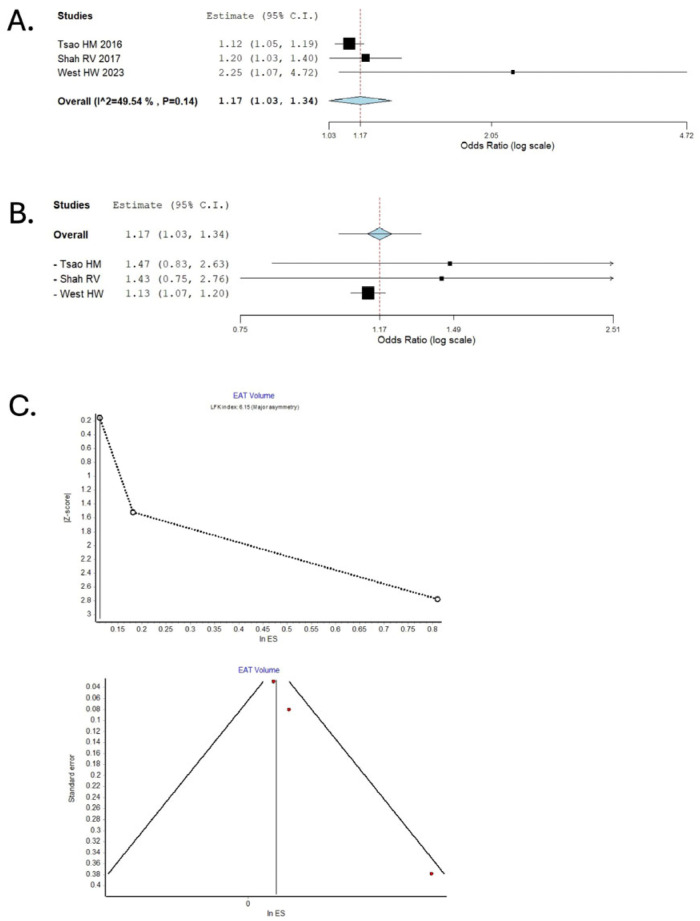
Association Between Epicardial Adipose Tissue Volume and Risk of Ischemic Stroke: Pooled Analysis and Assessment of Publication Bias. (**A**) Forest plot depicting random-effects model pooled data for the association between EAT volume and risk of ischemic stroke. (**B**) Sensitivity analysis by the leave-one-out method. (**C**) Publication bias assessed using DOI and Funnel Plots. In the forest plots, the size of each black square represents the weight of the corresponding study in the meta-analysis (larger squares indicate greater statistical weight). The center of each square denotes the study-specific effect estimate, and the horizontal line represents its 95% confidence interval. Red squares indicate the pooled effect estimate in sensitivity analyses.

Epicardial adipose volume (EAV) and its association with AIS were explored in three original studies: one cross-sectional (Tsao HM 2016), one prospective cohort (Shah RV 2017), and a secondary analysis of the SCOT-HEART trial (West HW 2023), which included 5907 patients [15,16,20]. Shah RV alone defined exposure as pericardial fat volume, including the epicardial fat volume, while the other two studies defined exposure as epicardial fat volume. Additionally, Tsao et al. focused exclusively on an atrial fibrillation population, further reinforcing the relevance of epicardial adipose volume (EAV) in this high-risk subgroup. In our pooled analysis of adjusted odds ratios, elevated EAV was associated with a 1.17-fold higher risk of acute ischemic stroke (AIS) (95% CI: 1.03–1.34), with moderate heterogeneity across studies (I^2^ = 49.5%, *p* = 0.14). Importantly, the robustness of this association was confirmed in leave-one-out sensitivity analyses; exclusion of the study by West et al.—which contributed the greatest statistical weight—did not materially alter the results, yielding a pooled aOR of 1.13 (95% CI: 1.07–1.20). Visual inspection of the funnel and DOI plots also suggested asymmetry, and the LFK index of 6.15, suggesting major asymmetry, highlights that these findings could also be limited by publication bias [Figure 4]. This pattern suggests that selective reporting of positive results may have led to a slight overestimation of the true pooled effect for EAT volume, and the risk estimate should therefore be interpreted conservatively. The three studies evaluating EAV demonstrated generally moderate methodological quality, with one study (West et al.) rated as low risk of bias due to its robust design and large sample size.

## 4. Discussion

The growing evidence linking EAT to adverse cardiovascular outcomes emphasizes its potential role in AIS pathogenesis, particularly among AFib patients. Understanding the predictors of AIS in this population is vital for optimizing management strategies. Our meta-analysis reinforces the association between increased EAT thickness and volume with heightened AIS risk, providing evidence of its role as a modifiable risk factor. Despite these findings, the underlying mechanisms remain multifaceted, with alternative explanations, such as comorbidities, genetic predispositions, and unaccounted lifestyle factors, necessitating consideration.

Recent evidence aligns with our findings. For instance, in a CT-based pooled meta-analysis of 26 cohorts, Chong et al. demonstrated a nearly two-fold increased risk of composite cardiovascular events among individuals with elevated EAT. However, stroke accounted for only ~4% of the reported endpoints, limiting event-specific conclusions. By contrast, our analysis is stroke-exclusive, thereby providing a more direct evaluation of the association between EAT and cerebrovascular risk [3]. Likewise, Hendricks et al. showed an adjusted HR of 2.1 for myocardial infarction in >6600 patients with elevated EAT. Taken together, our higher stroke-specific aOR (3.6) supports the hypothesis that EAT may exert a particularly potent pro-thrombotic influence on the cerebral vasculature [8].

EAT’s contribution to cardiovascular pathology is established. Through increased production of pro-inflammatory cytokines and pro-atherogenic mediators, it has been shown to negatively influence the myocardium [22,23,24]. Furthermore, it is independently associated with coronary artery disease and other metabolic diseases [23,24]. Iacobellis et al. identified elevated EAT levels in chronic AFib patients correlating with heart failure risk [25]. Similarly, Wang et al. demonstrated that individuals with acute myocardial infarction exhibited markedly higher EAT thickness compared with controls (5.6 ± 1.1 vs. 4.1 ± 1.0 mm; *p* < 0.001) [26]. Importantly, an EAT thickness exceeding 7 mm has been linked to a substantially increased risk of cardiovascular mortality, underscoring its prognostic relevance beyond arrhythmic and ischemic outcomes [27].

The relationship between Afib and AIS is firmly supported in the literature, and EAT may mediate this risk further via multiple pathways. Quantitative analyses, such as those from the Framingham Heart Study, demonstrate that pericardial fat volume is an independent predictor of AFib [28]. Studies have also demonstrated that EAT can be associated with paroxysmal and persistent Afib [29]. Further, periatrial EAT has been shown to independently predict AFib recurrence after catheter ablation, highlighting its direct impact on atrial remodeling and electrophysiological vulnerability [15].

Given the established role of AFib in embolic stroke, EAT’s association with AIS risk is both biologically plausible and clinically relevant [30]. While the biological plausibility of EAT contributing to AF-mediated embolism is strong, existing evidence remains partly conflicting. Some studies suggest that EAT primarily influences atrial remodeling and electrophysiological instability rather than direct thrombogenesis, while others highlight its central role in fostering pro-inflammatory and pro-thrombotic states [31,32]. Increased interleukin-1β secretion from EAT has been shown to reduce endothelial thrombomodulin expression, promoting thrombogenesis [33,34]. Studies by Bakirci et al. and Uslu et al. demonstrated that elevated epicardial adipose tissue (EAT) thickness was an independent predictor of intracoronary thrombus formation in patients presenting with NSTEMI and STEMI, respectively [34,35]. These observations are consistent with the broader concept that EAT contributes to a pro-thrombotic milieu through local inflammation, endothelial dysfunction, and impaired coronary microcirculation. Parallel to these mechanisms, our findings support the hypothesis that in atrial fibrillation patients, increased EAT burden may promote a thromboembolic predisposition, thereby conferring an elevated risk of AIS.

Monitoring EAT using accessible imaging modalities like echocardiography is crucial for integrating this marker into clinical practice. While computed tomography remains the gold standard for volumetric assessment, echocardiography offers a cost-effective, repeatable alternative that correlates with cardiovascular outcomes [36]. The CHA2DS2-VASc score is commonly employed for stroke risk stratification in AFib patients; combining it with EAT measurements could enhance predictive accuracy, as shown by Akdag et al. and Cosansu et al., who linked higher CHA2DS2-VASc scores with elevated EAT levels [18,37].

### 4.1. Clinical Implications

Our evidence indicates that an epicardial adipose tissue (EAT) thickness greater than 5 mm, as measured by transthoracic echocardiography, is significantly associated with an increased risk of ischemic stroke. This finding is clinically relevant because transthoracic echocardiography is a widely available, low-cost, and non-invasive tool, making EAT thickness a pragmatic biomarker that can be readily incorporated into routine cardiovascular risk assessment.

When considered alongside established risk stratification instruments such as the CHA_2_DS_2_-VASc score, the addition of EAT thickness may provide incremental prognostic value, particularly in atrial fibrillation patients who fall into intermediate- or borderline-risk categories where anticoagulation decisions are often challenging. Incorporating EAT into clinical decision-making could therefore refine patient selection for anticoagulation, potentially reducing both undertreatment and overtreatment.

Beyond arrhythmic and cerebrovascular risk, EAT also holds value in broader cardiovascular assessment. Zehir et al. demonstrated that EAT evaluation offers incremental diagnostic benefit in patients with high intermediate pre-test probability for coronary artery disease, supporting its role in refining cardiovascular risk stratification [38]. Integrating such evidence strengthens the translational relevance of our findings, suggesting that EAT quantification could guide more individualized preventive strategies.

However, despite the observed statistical significance of the association, the modest magnitude of the pooled effect estimate means that EAT’s clinical relevance lies not in its standalone power but strictly in its ability to provide incremental reclassification value over established risk scores. Integration into existing risk scores should be tested for improvements in net reclassification and calibration performance before being adopted into guidelines. If validated, EAT measurement could represent a simple yet powerful adjunct to current risk prediction models, bridging the gap between imaging-derived biomarkers and individualized stroke prevention strategies.

Finally, the COVID-19 pandemic underscored the close interplay between cardiovascular and pulmonary health. During this period, many patients experienced worsened cardiovascular outcomes secondary to pulmonary complications and systemic inflammation [39]. This “silent aspect” of the pandemic reinforces the importance of cardiovascular prevention, and EAT may serve as a crucial biomarker reflecting this systemic vulnerability to identify patients at heightened risk for both cardio- and cerebrovascular events.

### 4.2. Strengths and Limitations

Our study uses a robust and rigorous methodology, adhering to the MOOSE and PRISMA guidelines and employing standardized bias assessment tools to ensure the reliability and validity of the results. However, this meta-analysis has several caveats. First, although one randomized trial met our inclusion criteria, the remaining seven studies were observational (cohort, case–control or cross-sectional) and all carried a moderate risk of bias, so residual confounding—particularly by overall visceral adiposity—cannot be excluded. Second, only three cohorts reported volumetric CT or MRI measurements of epicardial adipose tissue, which reduced power for imaging-modality-specific analyses. Third, methodological diversity across designs contributed to substantial between-study heterogeneity (I^2^ > 70%) that persisted after leave-one-out sensitivity testing. Fourth, inspection of funnel and DOI plots revealed asymmetry consistent with small-study effects and possible publication bias. This risk may be magnified by our restriction to English-language articles, which, while necessary to ensure accurate data extraction and interpretation, may have introduced selection bias by excluding relevant studies in other languages. Fifth, because only eight studies met the inclusion criteria, we did not apply random-effects meta-regression or a formal GRADE profile; both require larger evidence bases to provide reliable estimates. Consequently, although the pooled association between elevated EAT and stroke appears robust, alternative explanations remain plausible, and prospective studies are needed to confirm these findings and refine clinical thresholds.

### 4.3. Future Directions

Prospective longitudinal cohort studies should further explore the association between EAT and risk of AIS in patients with AFib to establish temporality, while randomized controlled trials of interventions that lower EAT will be required to demonstrate causality. Additionally, there is value in exploring advanced imaging techniques for estimating EAT and the utility of point-of-care ultrasonography. This would allow the integration of EAT measurements into clinical practice to improve stroke risk stratification and guide management in the AFib population. Complementary avenues include (1) establishing large, prospective CT- or CMR-based registries that serially quantify EAT volume and adjudicate incident AIS, analyzed with robust causal-inference methods; (2) conducting randomized trials that target visceral-fat reduction—through intensive lifestyle programs, bariatric procedures, or pharmacotherapies such as GLP-1 receptor or SGLT2 inhibitors—with stroke end points and longitudinal EAT assessments; and (3) externally validating fully automated, artificial-intelligence segmentation algorithms across multi-vendor scanners to enable high-throughput, operator-independent EAT assessment. Collectively, these initiatives will convert associative observations into actionable, precision-based strategies for stroke prevention in the AFib population.

Moving beyond this robust meta-analytic evidence, the next critical step in this research field must be the transition from observational association to personalized predictive modeling and therapeutic intervention. We envisage the immediate future of this study to develop an external validation of a clinical risk score that incorporates EAT thickness/volume to enhance the predictive power of existing tools (like the CHA_2_DS_2_-VASc score for AF-related stroke). Also, we envision RCTs to test whether targeted interventions known to reduce EAT (e.g., specific pharmacological agents or intensive lifestyle modifications) result in a measurable reduction in the incidence of acute ischemic stroke.

**Figure 5:**
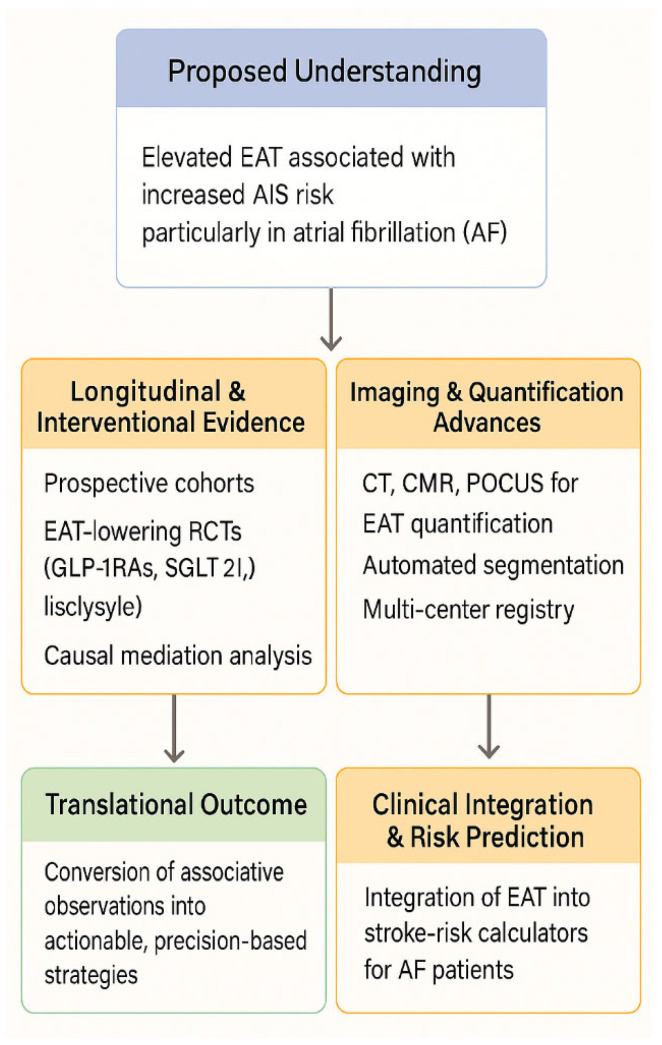
Proposed Future Research Pathways on Epicardial Adipose Tissue and Ischemic Stroke.

A visual summary of these proposed research priorities and translational pathways is presented in Figure 5.

## 5. Conclusions

In this meta-analysis of eight cohort studies comprising 7412 participants, both epicardial adipose tissue (EAT) thickness ≥ 5 mm and EAT volume > 120 cm^3^ were robustly associated with the risk of acute ischemic stroke (AIS). After adjustment for established clinical covariates—including age, sex, body mass index, CHA_2_DS_2_-VASc score, and atrial fibrillation status—the pooled analysis demonstrated an adjusted odds ratio of 3.6 (95% CI, 2.4–5.4) for AIS among individuals with elevated EAT compared to those with lower values.

Importantly, the direction and magnitude of the association were consistent across imaging modalities (transthoracic echocardiography, computed tomography, and cardiac magnetic resonance), suggesting that the prognostic signal is not confined to a single method of EAT assessment. Leave-one-out sensitivity analyses further confirmed the stability of the findings, with no single study exerting a disproportionate influence on the overall effect estimate.

Together, these results provide strong evidence supporting EAT as an independent and reproducible imaging biomarker of stroke risk, complementary to established clinical scoring systems.

## Figures and Tables

**Table 1:** Risk of Bias Assessment using Newcastle–Ottawa Scale and JBI Checklist for Randomized Controlled Trials.

Study	Number of Stars										Overall		
	Selection †			Comparability #				Exposure $					
Akıl E, 2014, Turkey [13]	**			**				*			5/9		
Altun I, 2016, Turkey [14]	***			**				*			6/9		
Tsao HM, 2016, Taiwan [15]	***			**				*			6/9		
Shah RV, 2017, USA [16]	**			**				*			5/9		
Cho KI, 2018, South Korea [17]	****			**				*			7/9		
Cosansu K, 2020, Turkey [18]	**			**				*			5/9		
Korkut M, 2022, Turkey [19]	***			**				*			6/9		
JBI-RCT Tool/Domains	D1	D2	D3	D4	D5	D6	D7	D8	D9	D10	D11	D12	D13
West HW, 2023, Scotland (RCT) [20]	Yes	UN	Yes	Yes	UN	Yes	Yes	UN	Yes	Yes	Yes	Yes	Yes

* indicates one point awarded in the Newcastle–Ottawa Scale; ** indicates two points (maximum scoring in the Comparability domain); *** indicates three points (maximum scoring in the Selection or Exposure/Outcome domains). **** Indicates four points (the maximum score) in the Selection domain of NOS. (†) Maximum four stars; (#) Maximum two stars; ($) Maximum three stars; JBI = Joanna Briggs Institute; RCT = Randomized Control Trial; UN = Unclear; D1 = Was true randomization used for assignment of participants to treatment groups? D2 = Was allocation to treatment groups concealed? D3 = Were treatment groups similar at the baseline? D4 = Were participants blind to treatment assignment? D5 = Were those delivering treatment blind to treatment assignment? D6 = Were outcomes assessors blind to treatment assignment? D7 = Were treatment groups treated identically, other than the intervention of interest? D8 = Was follow-up complete, and if not, were differences between groups in terms of their follow-up adequately described and analyzed? D9 = Were participants analyzed in the groups to which they were randomized? D10 = Were outcomes measured in the same way for treatment groups? D11 = Were outcomes measured in a reliable way? D12 = Was an appropriate statistical analysis used? D13 = Was the trial design appropriate, and were any deviations from the standard RCT design (individual randomization, parallel groups) accounted for in the conduct and analysis of the trial?

**Table 2:** Overview of Included Studies.

Author, Year, Country	Design	Mean Age Case/Control	Number of Cases (n) (F/M)	Number of Control (n) (F/M)	Definition of Exposure	Measurement of Exposure	Stroke Diagnosis	Size EAT	Size EAT
								Case	Control
Akıl E, 2014, Turkey [13]	Cross-sectional study	50.5 ± 13.9/ 53.7 ± 9.0	38 (15/23)	47 (20/27)	Epicardial Fat Thickness in mm	Echo TTE; Two measurements by two blinded cardiologists; Echo-free space between two layers of pericardium	CT and MRI	5.95 ± 1.14 mm	4.86 ± 0.68 mm
Altun I, 2016, Turkey [14]	Case–control study	71.4 ± 11/ 68.6 ± 8	61 (34/27)	82 (40/42)	Epicardial Fat Thickness in mm	Echo TTE; Echo-free space between the outer wall, myocardium and visceral pericardium	CT and MRI	4.8 ± 0.9 mm	3.8 ± 0.7 mm
Tsao HM, 2016, Taiwan [15]	Cross-sectional study	64.11 ± 11.43/ 63.25 ± 7.56	27 (8/19)	20 (5/15)	Epicardial Fat Volume	Contrast CT and EAT Volume	Clinical Signs	53.07 ± 14.67 cm^3^	21.46 ± 5.81 cm^3^
Shah RV, 2017, USA [16]	Prospective cohort	NR	NR	NR	Pericardial Fat Volume = Pericardial Fat Volume and Epicardial Fat Volume	CT and CMRI; Includes both Epicardial Fat and Pericardial Fat	NR	NR	NR
Cho KI, 2018, South Korea [17]	Retrospective cohort	Overall 65.4 ± 12.1/75.0 ± 10.6			Epicardial Fat Thickness in mm	Echo TTE; Two measurements by two blinded cardiologists; Echo-free space between the outer wall, myocardium and visceral pericardium	CT, MRI, CT-A, and MR-A	6.5 ± 1.2 mm	5.3 ± 1.2 mm
Cosansu K, 2020, Turkey [18]	Cross-sectional study	75.19 ± 9.39/ 73.72 ± 8.60	80 (50/30)	80 (46/34)	Epicardial Thickness in mm	Echo TTE; Measured twice by two cardiologists; Echo-free space between the outer wall, myocardium and visceral pericardium	CT and MRI	8.55 ± 1.08 mm	5.90 ± 1.35 mm
Korkut M, 2022, Turkey [19]	Case–control study	71.15 ± 12.32/ 69.78 ± 10.31	53 (25/28)	41 (22/19)	Epicardial Thickness in mm	Echo TTE; Three measurements, single-blinded physician; Echo: Free space between the RV and the inner sheet of the pericardium	CT and MRI	6.33 ± 1.47 mm	3.74 ± 0.61 mm
West HW, 2023, Scotland [20]	SCOT-HEART Randomized Control Trial	NR	NR	NR	Epicardial Fat Volume	CCTA Scans with DLN for Automated Estimation of EAT Volume	NR	NR	NR

NR = Not Reported; TTE = Transthoracic Echocardiogram; RV = Right Ventricle; CCTA = Coronary Computed Tomography Angiography; CT = Computed Tomography; MRI = Magnetic Resonance Imaging; EAT = Epicardial Adipose Tissue; DLN = deep-learning network; Cases = Defined as those with elevated epicardial adipose tissue thickness or volume as a categorical variable from each study.

## Data Availability

The data utilized in this meta-analysis were extracted from previously published studies. All data sources are publicly available and can be accessed through journals or databases. No new data were generated or collected for this study.

## Supplementary Materials

The following supporting information can be downloaded at https://www.mdpi.com/article/10.3390/medicina61122128/s1, Table S1: PRISMA 2020 Checklist [40].
